# Testicular histopathology and phosphorylated protein changes in mice with diabetes induced by multiple-low doses of streptozotocin: An experimental study

**Published:** 2018-04

**Authors:** Apichakan Sampannang, Supatcharee Arun, Jaturon Burawat, Wannisa Sukhorum, Sitthichai Iamsaard

**Affiliations:** 1 *Department of Anatomy, Faculty of Medicine, Khon Kaen University, Khon Kaen, Thailand.*; 2 *School of Medicine, Mae Fah Luang University, Chiang Rai 57100, Thailand.*; 3 *Center for Research and Development of Herbal Health Products, Faculty of Pharmaceutical Sciences, Khon Kaen University, Khon Kaen 40002, Thailand.*

**Keywords:** Diabetes mellitus, Phosphorylated protein, Streptozotocin, Mice

## Abstract

**Background:**

The streptozotocin (STZ)-induced diabetic model is widely used to evaluate the adverse effects of diabetes mellitus (DM) on spermatogenesis and testicular steroidogenesis. However, the actual mechanism of sub/infertility in DM males needs to be elucidated.

**Objective:**

To conduct a detailed examination of the testicular histopathology, sperm acrosome reaction (AR) status, and tyrosine-phosphorylated protein expression in the testis of male mice induced with STZ.

**Materials and Methods:**

Ten ICR mice were divided into two groups (n=5/each): control and diabetes induced by multiple low doses of streptozotocin (MLD-STZ). The control mice were intraperitoneally injected with citrate buffer, whereas MLD-STZ mice were injected with STZ at 40 mg/kg body weight for five consecutive days. At the end of the experiment (day 40), reproductive parameters, AR status, and the histopathology of the testis and epididymis were evaluated. The expression of testicular tyrosine phosphorylated proteins was examined.

**Results:**

Blood glucose levels, AR percentages, and sperm abnormality of STZ group were significantly higher (p=0.003, 0.001, 0.000), while sperm concentration was significantly lower (p=0.001) compared to control. Histopathology of the seminiferous tubule was classified into 7 types. Additionally, abundant round cells were found in the epididymal lumen of the MLD-STZ mice. Moreover, the intensities of testicular phosphorylated proteins (170, 70, 36, 30, and 25 kDas) were markedly higher and a 120 kDa protein band was noticeably lower in the MLD-STZ mice.

**Conclusion:**

MLD-STZ-induced DM causes many testicular histopathologies, precocious sperm AR, and increased expression of testicular phosphorylated proteins. These findings may clarify some mechanisms of sub/infertility in DM males.

## Introduction

Diabetes mellitus (DM), also called hyperglycemia, is one of the most prominent public health problems in modern societies and its incidence rate is elevating rapidly. The condition adversely affects the physiological functions of the spermatogenesis as well as testicular steroidogenesis and male fertility (1). These effects cause reductions in sperm quality in terms of attributes such as motility, viability, concentration, and normal morphology (2-3). Physiologically, the sperm acrosome reaction (AR) process is a specialized exocytotic event consisting of fusion and fenestration between sperm plasma and the outer acrosomal membrane resulting in the zona pellucida (ZP) being digested in order to facilitate successful sperm-ZP penetration during the early fertilization process (4). 

Premature or precocious AR is a cause of male infertility or impairs the quality of the sperm available for the process of fertilization. Additionally, it is well known that roles of tyrosine phosphorylated proteins are important for the regulation and coordination of various types of cell proliferation, division, growth, and differentiation in animals (5). It has also been demonstrated that tyrosine phosphorylated proteins are involved in sperm production, capacitation, and AR in post-translational modifications in the male reproductive system (6). Such phosphorylated proteins have only been localized in the Sertoli cells and late spermatids, which might play a role in spermatogenesis, especially process of the spermiogenesis in testis (7). It is well known that protein phosphorylation is essential for sperm capacitation and acrosome reaction leading to male infertility by impairment of sperm function (8-10). In addition, previous studies have demonstrated that the patterns of testicular phosphorylated proteins are changed under different inductions (drugs, stressors, or chemotherapeutic reagents), which involved in increasing levels of epididymal sperm acrosome reaction and semen abnormalities (6, 11-14). Absolutely, these findings are causes of male infertility. However, alterations of sperm physiology and other potential markers in the testis that lead to male infertility in DM conditions require further study. Previous investigators have not elucidated the mechanisms of diabetes induced by multiple low doses of streptozotocin (MLD-STZ)-induced DM's adverse effects on male reproduction in animals, especially with regard to AR status and patterns of phosphorylated protein expressions in the testicular lysate. 

Therefore, this study endeavored to study the histopathologies of testis and epididymis, epididymal sperm AR status, and expression patterns of testicular phosphorylated proteins in MLD-STZ induced DM mice in order to describe the actual mechanisms of male reproductive dysfunction resulting from DM.

## Materials and methods

**Animals and diabetic induction**

ICR male mice (30-40 gr) were purchased from the National Laboratory Animal Center, Mahidol University, Salaya, Nakhon Pathom province, Thailand. Animals were housed in stainless steel cages under controlled environmental conditions (temperature 22C±2^o^C, 12 hr light/dark cycles, Relative humidity 30-60%, sound <85 decibels, light intensity 350-400 lux). All mice received commercial food pellets daily and water ad libitum. Ten ICR mice were divided into control and MLD-STZ (multiple-low doses of streptozotocin) groups (five mice in each group). In the control group, mice were intraperitoneally injected with 0.1 M citrate buffer (pH=4.5), whereas in the MLD-STZ group, mice were injected with STZ (dissolved in citrate buffer) at 40 mg/kg body weight for five consecutive days. All mice were weighed daily throughout the 40 days of the experiment. After STZ induction (days 8, 12, 19, 26, 33, and 40), blood was gathered from a tail prick after fasting and the glucose levels of the mice were measured using a blood glucose oxidase reaction monitoring system (Johnson and Johnson Ltd., USA). Mice were considered to be diabetic when their blood glucose levels were greater than 250 mg/dl.

**Morphological and histopathological studies of the male reproductive organs**

After euthanasia, male reproductive organs (testis, vas deferens plus epididymis, seminal vesicle plus prostate gland, and penis) were collected and weighted. The absolute weights of the organs were calculated and expressed as relative weight (g/100g). These organs were then observed and photographed using a digital camera (Nikon Coolpix S2600, Japan). For histopathological examination, the right testis and epididymis were fixed in 10% formalin (pH=7.4) for two days. These tissues were then routinely processed for examination by light microscope. The paraffinized-tissue blocks were subsequently sectioned at 5-7 µm and stained with hematoxylin and eosin (H & E). The histopathological images were photographed using a Nikon light ECLIPSE E200 microscope equipped with a DXM1200 digital camera.

**Sperm count and morphology**

The left caudal epididymis and vas deferens were operated on and gently squeezed to collect sperm fluid. The epididymal sperm fluid was dipped and suspended in 1 ml of phosphate-buffered saline (PBS) at 37^o^C, pH=7.4). The diluted sperm suspension was subsequently centrifuged at 5,000 rpm, 25^o^C for 2 min in order to wash and separate the mature sperm pellet from its fluid. Then the sperm pellet was re-suspended with 1 ml PBS. The sperm suspension was subsequently diluted with PBS (1:20 dilution) before counting. The diluted sperm suspension (10 µl) was placed in a Neubauer counting chamber and the sperm was counted under a light microscope (Nikon ECLIPSE E200, Japan) in triplicate. In order to examine sperm morphology, the diluted sperm was smeared on a glass slide and dried overnight in a hot-air oven set at 50^o^C. 

The dried sperm was then fixed with methyl alcohol for 20 min and stained with hematoxylin (20 min) and eosin (5 min). After washing, the stained slide was dehydrated with an ascending series of alcohols. Types of normal and abnormal sperm morphologies were classified as previously described (15). In the classification of sperm abnormalities, we have used criteria based on Monika and Ward (2005). As previously described, head and tail abnormal morphologies were classified into 8 types; 1) thin-elongated head (H1), 2) club-shaped head (H2), 3) mild head defects (H3), 4) bent head (T1), 5) looping- mid piece (T2), 6) folded mid- and principal piece (T3), 7) incorrect head-neck connection (T4), 8) lasso-like (T5), respectively (15). In each mouse, 200 sperms were counted and used to calculate the total percentage of abnormal sperm morphology.

**Sperm acrosome reaction assay**

In order to assess the sperm acrosome reaction, the diluted-sperm suspension was gently smeared on a gelatin-coated slide. Then the sperm was air dried at room temperature for 24 hr. The dried sperm was subsequently stained with 0.22 % Coomassie blue for 5 min. After washing, the stained sperm slide was mounted with mounting media. Sperm acrosomes were classified into two types as previously described (11, 14). Sperm presenting an acrosome cap stained with Coomassie blue were classified as acrosome intact, whereas sperm without staining on the cap was classified as AR. Two hundred total sperm were counted and the percentage of acrosome-reacted sperm was calculated.

**Immuno-Western blotting analysis of tyrosine phosphorylated protein expressions**

The testicular tissue was homogenized with Radioimmunoprecipitation assay buffer (Cell Signaling Technology, Inc., USA) containing a cocktail of protease inhibitors (Sigma, Inc., USA) and centrifuged at 12,000 rpm at 4^o^C for 10 min to separate testicular soluble proteins from the pellet. The total testicular protein concentration was measured using a NanoDrop spectrophotometer (ND-1000 NanoDrop Technologies, Inc., USA) at an absorbance of 280 nm. Testicular protein lysate (100 µg/ lane) was separated on 12% sodium dodecyl sulfate–polyacrylamide gel electrophoresis (SDS-PAGE). 

Separated proteins were transferred onto the nitrocellulose membrane. The membrane was subsequently incubated with 5% skim milk in 0.1% phosphate-buffered saline/Tween (PBST) (0.1% Tween-20, PBS, pH=7.4) for 1 hr and individually incubated with anti-phosphotyrosine primary antibody (1:1,000; Millipore Co., USA) or β-actin antibody (1:1,000 dilution; Santa Cruz Biotechnology, Inc., USA) at 4^o^C overnight. Then the membrane was washed in 0.05% PBST (0.05% Tween-20, PBS, pH=7.4) for 5 min (three times) and incubated with specific secondary antibody conjugated with goat anti-mouse IgG (1: 2,000 dilutions) for anti β-actin for 1 hr at room temperature. 

The membrane was washed with 0.05% PBST before detection of target proteins using enhanced chemiluminescence substrate under ImageQuant 400 (GH Healthcare, USA). An EGF stimulated A413 cell lysate or bovine albumin serum was used as a positive or negative control. In terms of quantitative analysis, the relative intensities of phosphorylated protein patterns were measured and analyzed using ImageJ software version 14.9.

**Ethical consideration**

This experiment was approved by the Animal Ethics Committee of Khon Kaen University, based on the Ethics of Animal Experimentation as determined by the National Research Council of Thailand (ref. No. 0514.1.12.2/35 with record No. AEKKU-NELAC 29/2557).

**Statistical analysis**

All data were represented as a mean±standard deviation. An independent t-test was performed to examine the significant differences between the control and MLD-STZ groups using SPSS (Statistical Package for the Social Sciences, version 19.0, SPSS Inc, Chicago, Illinois, USA) software. The mean difference is significant at the 0.05 level (p<0.05).

## Results

**Effects of MLD-STZ on blood glucose levels and body weight**

**Figure 1:**
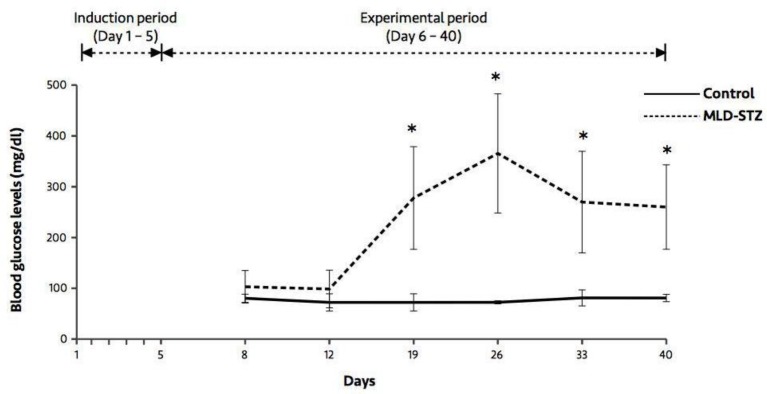
Blood glucose levels in the control and MLD-STZ groups. Data are represented as mean±SD (n=5/each group). *p<0.05

**Figure 2:**
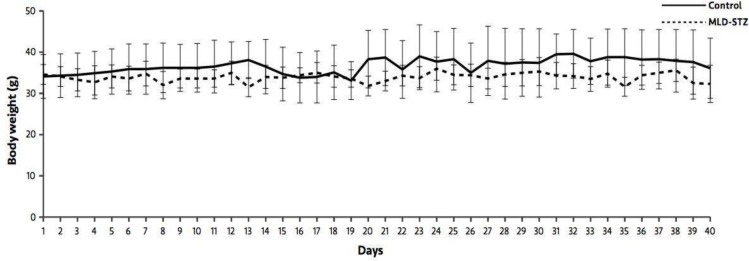
Body weight in the control and MLD-STZ groups for 40 experimental days.

At days 19, 26, 33, and 40, the results showed that blood glucose levels in the MLD-STZ group were significantly higher (p=0.005, 0.012, 0.019, 0.007) than those of the control group (Figure 1). As shown in Figure 2, there were no significant differences in body weight between the control and MLD-STZ groups, which was compared daily for 40 consecutive days.

**Effect of MLD-STZ on male reproductive organ weight, sperm concentration, and sperm AR status**

**Figure 3:**
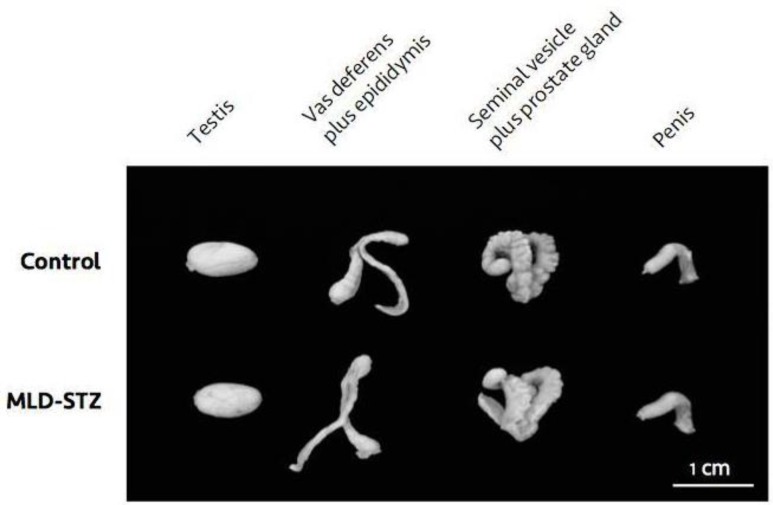
Representative morphological photographs of the testis, vas deferens plus epididymis, seminal vesicle plus prostate gland, and penis as compared between the control and MLD-STZ groups

During morphological observation, the testis, vas deferens plus epididymis, seminal vesicle plus prostate gland, and penis of MLD-STZ mice showed no difference in terms of size than those of the control (Figure 3). In addition, there was no significant difference in the absolute and relative weights of the organs between the two groups (Table I). However, sperm concentration in the MLD-STZ group was significantly lower (p=0.001) than that of the control group (Table I). Furthermore, the percentage of acrosome-reacted sperm in the MLD-STZ group was significantly higher (p=0.001) than that of the control (Table I).

**Effect of MLD-STZ on epididymal sperm morphology**

**Figure 4.**
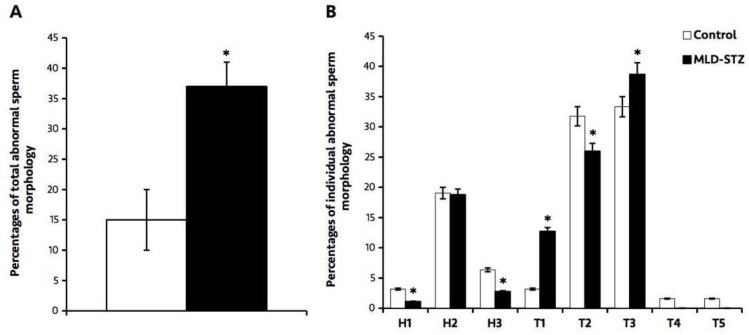
The percentages of the total (A) and individual abnormal sperm morphology compared between the control and MLD-STZ groups. Head abnormalities: H1, thin-elongated head; H2, club-shaped head; H3, mild head defects. Tail abnormalities: T1, bent head; T2, looping mid- piece; T3, folded mid- and principal piece; T4, incorrect head-neck connection; T5, lasso-like.. Data point represented as mean±S.D. (n=5each group). *p<0.05

Abnormal sperm morphology observed in this study was classified into eight individual types (Figure 4B). The results showed that the total percentage of abnormal sperm morphology in the MLD-STZ group was significantly higher (p=0.000) than that of the control group (Figure 4A). In particular, there were significant (p=0.015, 0.012, 0.000) decreases in sperm with thin-elongated heads (H1), mild head defects (H3), and looping-mid pieces (T2), as well as significant (p=0.005, 0.000) increases in sperm with tail bent heads (T1) and tail folded mid- and principal pieces (T3) in the MLD-STZ group as compared to the control group (Figure 4B). We found no significant difference in the percentages of sperm club-shaped heads (H2), sperm with incorrect head-neck connections (T4), or lasso-like sperm (T5) between the two groups (Figure 4B).

**Effect of MLD-STZ on histopathology of the testis and epididymis**

**Figure 5:**
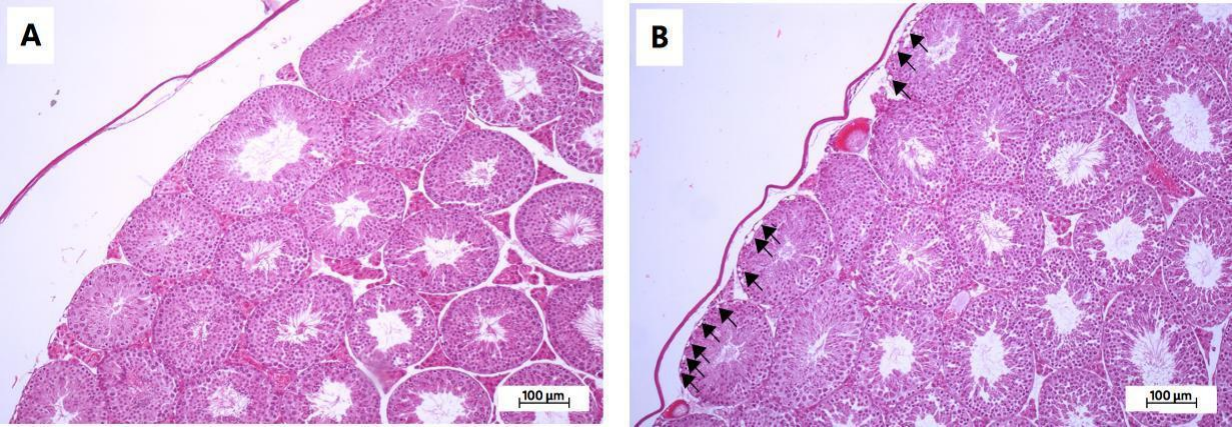
Representative photomicrographs showing histopathology (H&E) of the testis in the control (A) and MLD-STZ (B) groups. Arrows indicate vacuolation in the seminiferous epithelium

**Figure 6:**
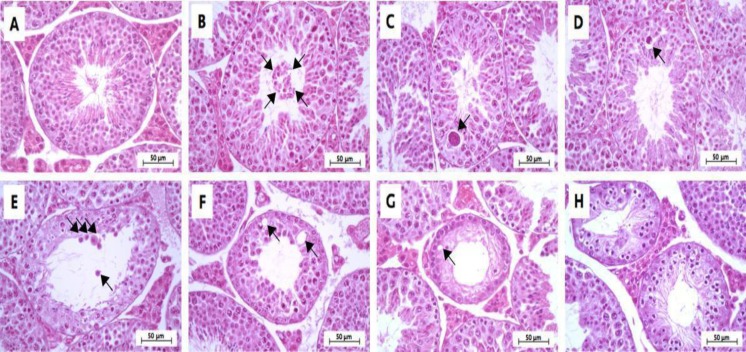
Representative photomicrographs showing normal histology of the control group (A) and histopathology of seminiferous tubules found in the MLD-STZ group (B-H). Normal arrangement of spermatogenic and Sertoli cells (A), sloughing of deciduous and spermatogenic cells (arrows) into the tubular lumen , large-sized nucleus or bi-nucleated cells (arrow) in the seminiferous epithelium (C), small-sized nucleus cells with vacuolization (arrow) in the seminiferous epithelium (D), few layers of spermatogenic cells and artifactual sloughing (arrows) of germ cell elements into the lumen (E), vacuolation (arrows) of Sertoli cell and absence the spermatids (F), atrophy with germ cell degeneration and small vacuolization (arrow) between spermatogonia and Sertoli cells (G), and the hypospermatogenesis with all germ layers diminished (H

**Figure 7:**
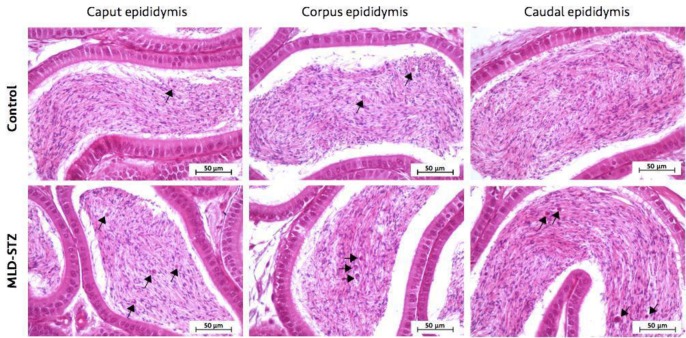
Representative photomicrographs showing the histopathology of caput, corpus, and caudal epididymis in the control and MLD-STZ groups. Arrows indicate round cells found in the sperm mass of the epididymal lumen

Investigation of the histopathology of the testis (Figure 5), seminiferous tubule (Figure 6), and epididymis (Figure 7) revealed the obvious presence of vacuolation in the seminiferous epithelium in the MLD-STZ group (Figure 5B). Additionally, histopathology of the seminiferous tubules found in the MLD-STZ group was classified into seven types (Figure 6B-H) as follows: sloughing of deciduous and spermatogenic cells into the tubular lumen (Figure 6B), large-sized nucleus or bi-nucleated cells in the seminiferous epithelium (Figure 6C), small-sized nucleus cells with vacuolization in the seminiferous epithelium (Figure 6D), few layers of spermatogenic cells and artifactual sloughing of germ cell elements into the lumen (Figure 6E), vacuolation of Sertoli cell and absence the spermatids (Figure 6F), atrophy with germ cell degeneration and small vacuolization between spermatogonia and Sertoli cells (Figure 6G), and hypospermatogenesis with all germ layers diminished (Figure 6H). The rates of these histopathological types in the MLD-STZ group are compared to those of the control in Figure 6A. Furthermore, the lumens of caput, corpus, and caudal epididymis in the MLD-STZ groups showed round cells as compared to the control group (Figure 7).

**Effect of MLD-STZ on expression patterns of testicular tyrosine phosphorylated proteins**

**Figure 8:**
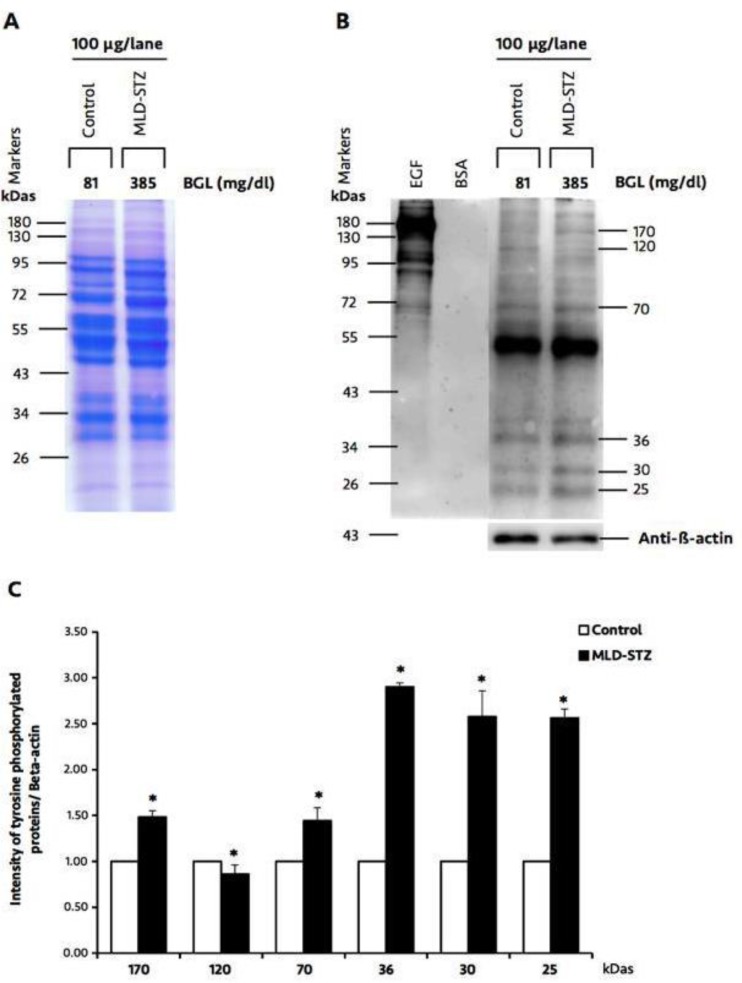
Representative testicular protein profile revealed by SDS-PAGE (A), immuno-Western blotting of testicular phosphorylated proteins, and relative intensity of testicular phosphorylated proteins (170, 120, 70, 36, 30, and 25 kDas; C) compared between the control and MLD-STZ groups. Epidermal growth factor (EGF)-like factor and bovine serum albumin (BSA) used as positive and negative controls for phosphotyrosine antibody. β-actin was used as an internal control. BGL: blood glucose levels

The tyrosine phosphorylated protein patterns of the testicular lysate are shown in Figure 8B. The total equal amount loading proteins were confirmed using SDS-PAGE (Figure 8A). 

Although positive protein patterns looked similar in both groups, only six major protein bands (170, 120, 70, 36, 30, and 25 kDas) were differentially expressed in each group (Figure 8B). The relative intensity of 170, 70, 36, 30, and 25 kDas phosphorylated proteins in MLD-STZ group was significantly increased as compared to than that of control. In contrast, the expression of a phosphorylated 120 kDa protein was decreased in MLD-STZ group as compared to the control (Figure 8C).

**Table I:** Comparisons of male reproductive organ weight, sperm concentration, and acrosome-reacted sperm between the control and MLD-STZ groups

**Parameter**	**Control** ** groups**	**MLD-STZ** ** groups**
Testis		
Absolute weight (g)	0.14 ± 0.02	0.14 ± 0.01
Relative weight (g/100g)	0.39 ± 0.05	0.42 ± 0.02
Vas deferens plus epididymis		
Absolute weight (g)	0.07 ± 0.01	0.07 ± 0.01
Relative weight (g/100g)	0.19 ± 0.02	0.20 ± 0.02
Seminal vesicle plus prostate gland		
Absolute weight (g)	0.27 ± 0.09	0.20 ± 0.06
Relative weight (g/100g)	0.75 ± 0.24	0.61 ± 0.18
Sperm concentration (10^6^cells/ml)	13.09 ± 0.73	8.25 ± 0.63*
Acrosome-reacted sperm (%)	4.50 ± 2.20	11.10 ± 4.00*

MLD-STZ: Multiple-low doses of streptozotocin

* Significant difference (p<0.05). Data are represented as mean±S.D. (n=5/each group).

Data are represented as mean±SD (n=5/each group).

*p<0.05

## Discussion

In this study, the results demonstrated that DM mice induced by MLD-STZ has severity of testicular and epididymal histopathologies. In addition, STZ adversely affected sperm parameters including concentration, AR, and morphology. Especially, it could change expression pattern of tyrosine-phosphorylated proteins in testicular tissue.

DM is one cause of male infertility (1). In animal models, streptozotocin (STZ), a diabetogenic agent, has been widely used for DM induction in order to investigate the actual mechanism of various metabolic syndromes including male infertility (16-18). Recently, MLD-injections or one high dose (OHD)-STZ injection are commonly used to induce type I DM in animal models (16-17). Although both methods can induce insulin deficiency and hyperglycemia, MLD-STZ inductions have a low frequency of mortality before the end of the experiment and can more closely mimic human DM physiogenesis than OHD-STZ injection (16-17). This study was performed on animals induced with type 1 DM by MLD-STZ in order to investigate the actual mechanisms of DM infertile males.

This study showed a reduction of sperm concentration in MLD-STZ mice, which is similar to the results of previous investigations in MLD-STZ rats and mice (19-20), as well as those who underwent OHD-STZ mice model (16). This reduction may be caused by low testosterone levels and decreases in total Leydig cell numbers (2, 21). It has also been shown that STZ injection affects spermatogenesis impairments by decreasing follicle stimulating hormone and luteinizing hormone levels and disturbing Sertoli cell function (2, 21-22). Some previous studies have demonstrated reductions in testicular, epididymal, and seminal vesicle weight in OHD-STZ-treated animals (16, 23). This effect corresponds to a reduction of testosterone impairment (2, 21). In contrast to previous studies, we found no statistical difference in terms of reproductive organ weight between the MLD-STZ and control groups. Unfortunately, testosterone levels in this study were not enough to be measured in order to explain why organ weight had not decreased. 

The increases in total abnormal sperm morphology found in this study are similar to the findings of previous studies of OHD-STZ animal models (3, 16). A previous study found over expressions of the Cnot7 gene in the livers of mice induced with obesity through a high-fat diet (24). This gene has also been found to be expressed in Sertoli and Leydig cells (25). As reductions in Cnot7 gene expression can cause abnormalities of sperm morphology and DM can cause Sertoli cell dysfunction, we assume that sperm abnormality in MLD-STZ mice may be associated with low expression of this gene being involved in Sertoli cell impairment (22, 26). This impairment may interfere with spermiogenesis, resulting in abnormal sperm production from spermatid. However, expressions of the Cnot7 gene in DM-induced via STZ should be investigated further in order to clarify the association.

Premature or precocious sperm AR can be a cause of male infertility (6, 11-14, 27-28). A previous study showed that precocious sperm AR stained by calcium ionophore A23187 challenge, and lectin was increased in OHD-STZ induced DM rats (27-28). In addition, increases in AR sperm have been found in rats exposed to immobilization stress (9). Although the AR status of epididymal sperm has been previously reported in OHD-STZ animal models (27-28), this study is the first to report this in MLD-STZ-treated mice. Levels of testicular nerve growth factor found in Sertoli cells and early spermatids were significantly decreased in the DM animals (29-30), which implies that this deficit is involved in AR formation impairment and that DM affected the regulation of some signaling pathways in the testis via the nerve growth factor-mitogen-activated protein kinase signaling pathway (30-31). A recent study described the histopathology of seminiferous tubules in MLD-STZ mice for the first time. The observed tubular histopathology was classified into seven types (Figure 6B-H). The intraepithelial vacuolization found in this study is similar to that of previous studies in OHD-STZ rats (32-34). This type of histopathology is one of the most common injuries in Sertoli cells (35). Furthermore, the decreases in seminiferous tubule epithelium diameter found in this study were similar to those found in OHD-STZ rats (3, 32, 36). 

Atrophy with germ cell degeneration has also been observed in OHD-STZ rats, which is consistent with in our results (in MLD-STZ mice) (32-33). This degeneration is described by the functional interruptions of Sertoli cells (35). Our study found both small, large, and bi-nucleated cells in seminiferous epithelium, which is consistent with the results of a study that found multinucleated giant cells in OHD-STZ rats (32). It is possible that this is due to the failure of cytokinesis during meiotic division especially nondisjunction in chromosomes (37). Additionally, the germ-cell exfoliation found in this study is similar to that which has been previously found in an OHD-STZ rat model (32). This alteration is involved in the loss of premature spermatogenic cell adhesion to Sertoli cell processes (38). Such changes also caused artifactual sloughing of germ cell elements into the tubular lumen, which was also found in a study by Donmez and colleagues (32, 38). This study also found hypospermatogenesis, which has also been found in OHD-STZ rats (39). A previous study found immature round cells, degenerated sperm heads and residual bodies in both caput and caudal epididymal lumens in OHD-STZ rats (34). In MLD-STZ mice, abundant round cells in caput, corpus, and caudal epididymal lumens were observed in our study. This may result from germ cell loss from seminiferous epithelium and testosterone impairment (35, 38).

Tyrosine phosphorylated proteins have been localized within the Sertoli cells and late spermatids of the testes (7). They are assumed to be involved in spermatogenesis, sperm capacitation, and acrosome reaction (6, 11-14). Interestingly, the relative intensities of testicular 170, 70, 36, 30, and 25 kDa phosphorylated proteins in this study were markedly increased, while that of 120 kDa protein was noticeably low in MLD-STZ mice. In contrast, expression patterns of 66 and 50 kDa proteins have similarly been observed in both OHD-STZ and control rats (2). Although a previous study showed two bands of tyrosine phosphorylated proteins in OHD-STZ rats (2), this is the first study to report changes to different six phosphorylated proteins in mice with MLD-STZ-induced DM. It is possible that this is due to differences in DM induction and animal strain models. These proteins may play a role in spermatogenesis and acrosome formation. However, these testicular phosphorylated proteins should be investigated further in order to determine their actual structure and function.

## Conclusion

In conclusion, DM could increase percentages of abnormal sperm morphology and sperm acrosome reaction and reduce sperm production in an animal model. Although we could not definitively illustrate alterations in the patterns of testicular 170, 120, 70, 36, 30, and 25 kDas phosphorylated protein expression in mice with MLD-STZ-induced DM, we suggest that post-translational modifications to those proteins might correlate with suppression of spermatogenesis, spermiogenesis, or acrosome formation due to phosphorylated proteins having been localized in the Sertoli cells and late spermatids (with the exception of Leydig cells). Moreover, such alterations in the patterns of these proteins might be correlated with occurrences of histopathological lesions in MLD-STZ mice.
